# Description of a Novel Mechanism Possibly Explaining the Antiproliferative Properties of Glucocorticoids in Duchenne Muscular Dystrophy Fibroblasts Based on Glucocorticoid Receptor GR and NFAT5

**DOI:** 10.3390/ijms21239225

**Published:** 2020-12-03

**Authors:** Sandrine Herbelet, Boel De Paepe, Jan L. De Bleecker

**Affiliations:** 1 Department of Neurology, Ghent University Hospital, Ghent University, C. Heymanslaan 10, 9000 Ghent, Belgium; Boel.DePaepe@UGent.be (B.D.P.); Jan.DeBleecker@UGent.be (J.L.D.B.); 2 Neuromuscular Reference Center, Ghent University Hospital, C. Heymanslaan 10, 9000 Ghent, Belgium

**Keywords:** Duchenne muscular dystrophy fibroblasts, methylprednisolone, hydrocortisone, reduction in proliferation, glucocorticoid receptor, NFAT5

## Abstract

Glucocorticoids are drugs of choice in Duchenne muscular dystrophy (DMD), prolonging patients’ ambulation. Their mode of action at the protein level is not completely understood. In DMD, muscle tissue is replaced by fibrotic tissue produced by fibroblasts, reducing mobility. Nuclear factor of activated T-cells 5 (NFAT5) is involved in fibroblast proliferation. By treating one DMD fibroblast cell culture and one of unaffected skeletal muscle fibroblasts with methylprednisolone (MP) or hydrocortisone (HC) for 24 h or 12 d, the antiproliferative properties of glucocorticoids could be unraveled. NFAT5 localization and expression was explored by immunocytochemistry (ICC), Western blotting (WB) and RT-qPCR. NFAT5 and glucocorticoid receptor (GR) colocalization was measured by ImageJ. GR siRNA was used, evaluating GR’s influence on NFAT5 expression during MP and HC treatment. Cell proliferation was monitored by IncuCyte ZOOM. In DMD fibroblasts, treatment with MP for 24 h induced dots (ICC) positive for NFAT5 and colocalizing with GR. After 12 d of MP or HC in DMD fibroblasts, NFAT5 expression was decreased (RT-qPCR and WB) and growth arrest was observed (Incucyte ZOOM), whereas NFAT5 expression and cell growth remained unchanged in unaffected skeletal muscle fibroblasts. This study may help understand the antiproliferative properties of glucocorticoids in DMD fibroblasts.

## 1. Introduction

The major mode of action of glucocorticoids (GCs) was described in 1994 by Ray and Prefontaine [1]: the glucocorticoid receptor GR binds to the master regulator of inflammation nuclear factor kappa-light-chain-enhancer of activated B cells (NF-κB). GCs induce reduced cell proliferation by cell cycle arrest in the G1-S phase [2,3]. Cortisol is the natural GC produced in the body in the cortex of the adrenal glands, during the acute stress response [4,5]. Many synthetic GCs are used in clinics such as beclomethasone, betamethasone, cortisone, dexamethasone, fluticasone, hydrocortisone (HC), methylprednisolone (MP), prednisolone and triamcinolone. MP is produced as methylprednisolone sodium succinate (MPSS) for better dissolution in plasma. However, after hydrolysis in the liver, only a fraction of MP remains available for therapeutic action. Besides, 75% of intravenous (IV) MP binds in plasma to albumin and corticoid binding globulin (CBG), also known as transcortin. This results in a nonlinear relationship between free and bound MP and an impact on MP bioavailability [6]. Several studies have been performed with different IV doses and report superimposable concentration curves for total MP in plasma following different IV doses [7]. A range of 0.1–8 µg/mL total MP in plasma at time point +0.5–18 h after IV or IM MP injection is found across the studies, independently of the used dose [7,8,9]. Free MP concentration in plasma varies in a range of 0.001–1 µg/mL plasma. Cell culture media are often supplemented with bovine serum naturally containing albumin and transcortin. MP hydrolysis has also been described in cell culture media: 10–43% of the drug is hydrolyzed in 2–4 days [10,11]. Hydrocortisone (HC) is produced as hydrocortisone sodium succinate (HCSS) and is very similar to endogenous cortisol. Up to 78% is bound in plasma [6]. Ranges from 0.39 to 1.21 µg/mL total cortisol in plasma and 0.05–0.39 µg/mL of free cortisol in plasma have been measured from time point + 0.5–5 h after IV administration [12].

Corticoids are also drugs of choice in Duchenne muscular dystrophy (DMD) [13]. Mutations in the dystrophin gene are the genetic cause of the inherited muscle disease DMD [14,15], characterized by chronic inflammation and hampered muscle regeneration [16]. The major debilitating factor in DMD is the formation of fibrotic scar tissue [17], which is an excess of extracellular matrix formation (ECM), especially collagen. Two major components induce fibrosis: chronic inflammation and chronic proliferation of fibroblasts [18].

In the first stage of muscle repair, fibroblasts provide essential temporary scaffolding elements to new muscle fibers and neuromuscular junctions such as laminin, proteoglycans, fibronectin, elastine and collagen types I and III [18]. This scaffold may become a permanent and impairing secondary tissue when inflammation becomes persistent after dysregulation in the ratio of the macrophages M1/macrophages M2 [19]. Mouse embryonic fibroblasts lose their proliferative capacity when the nuclear factor of activated T-cells 5 (NFAT5) is inhibited [20,21]. In a previous study, abnormal NFAT5 physiology was found in DMD fibroblasts from one patient. Both DMD and unaffected fibroblasts reached confluency around 15 days. Unaffected skeletal muscle fibroblasts showed a growth factor of 12–15 compared to control levels, with confluency around 80%. NFAT5 was merely located in the nucleus and did not respond to proinflammatory cytokines. DMD fibroblast growth was not hampered by this proinflammatory environment. This observation could explain permanent fibrotic matrix production in DMD [22]. NFAT5 is known as a member of the Rel family of transcription factors and harbors similar elements of NF-κB, which is inhibited by GCs by trapping it in cytoplasmic complexes [23,24].

In this study, one DMD (male, 11 years old) and one cell culture from unaffected skeletal muscle fibroblasts (male, 17 years old) used in a previous study [22] were treated with HC or MP, expecting an influence of the GCs treatment on NFAT5 localization and expression in DMD fibroblasts and a possible change in fibroblast proliferation over time. With GCs trapping NF-κB in the cytoplasm, the interaction between NFAT5 and GR was investigated.

## 2. Results

### 2.1. Hydrocortisone and Methylprednisolone only Decrease DMD Fibroblasts Growth

**Figure 1:**
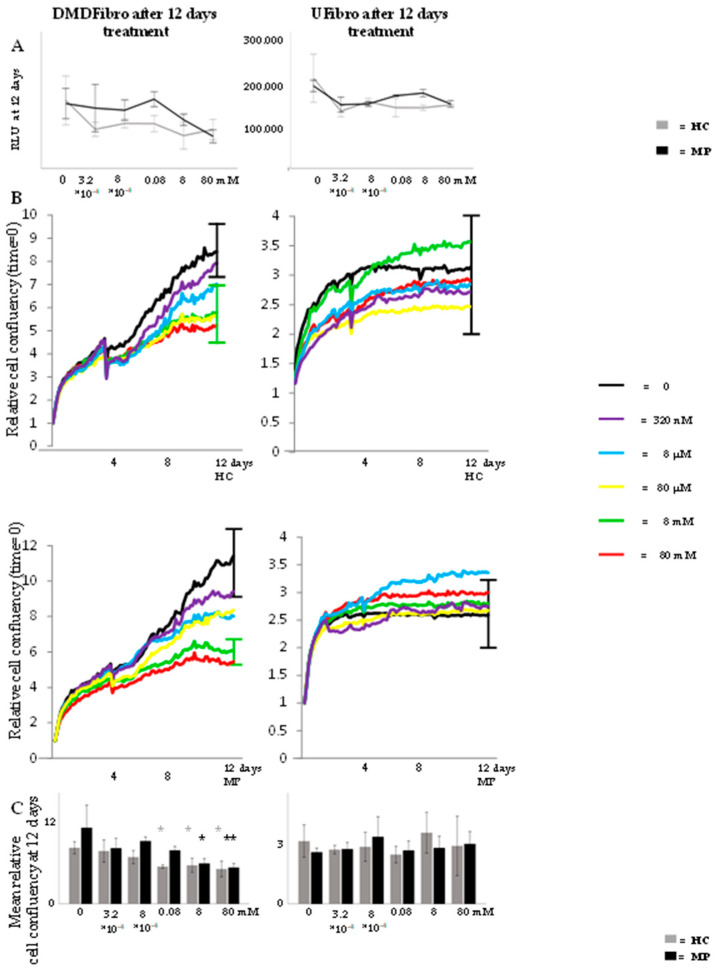
Cell viability and relative cell confluence in Duchenne muscular dystrophy (DMD) and unaffected fibroblasts treated for 12 d with hydrocortisone (HC) or methylprednisolone (MP). Both in DMDFibro and UFibro, all cells keep their viability, translated in relative light units (RLU at 12 d) (**A**). Only DMDFibro shows decreased relative cell confluence as measured by Incucyte ZOOM (**B**) after treatment with HC (right panel in (**B**)) and MP (left panel in (**B**)). After statistical analysis of relative cell confluency in DMDFibro and UFibro treated for 12 d with HC or MP (**C**), significance was observed in DMDFibro after HC or MP treatment (left histogram in (**C**)); * = *p* < 0.05 and ** = *p* < 0.01; *n* = 3).

**Figure 2:**
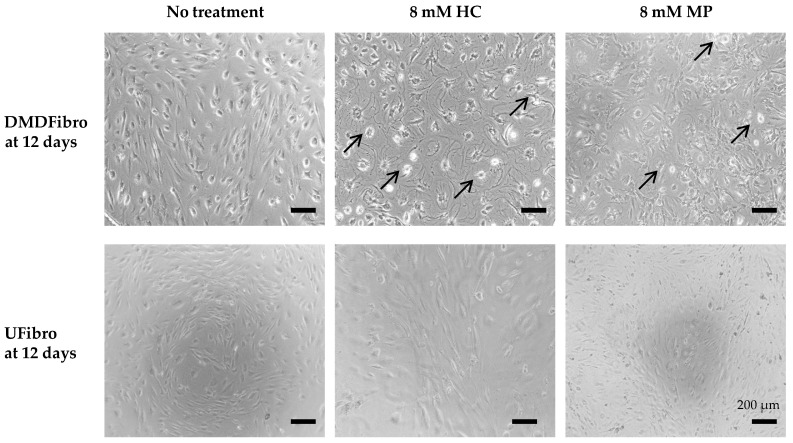
Cell morphology in DMD and unaffected fibroblasts treated for 12 d with hydrocortisone (HC) or methylprednisolone (MP). In DMDFibro, cells show detachment (black arrows) and shrinkage after treatment with 8 mM HC and 8 mM MP for 12 days. At day 12, UFibro show 80% cell confluency.

Both DMD fibroblasts (DMDFibro) and unaffected fibroblasts (UFibro) remained viable during the entire 12 d treatment with HC or MP ranging from 80 nM (0.025 ng/mL) to 80 mM (25 µg/mL). No statistical difference could be shown in DMDFibro viability despite differences visible in cell morphology under the phase contrast microscope after 12 days of treatment (Figure 1A and Figure 2). DMDFibro showed a statistically significant decrease in cell confluency when treated with 0.08 mM HC, 8 mM HC, 80 mM HC or 8 mM MP (*p* < 0.05). Treatment with 80 mM MP for 12 d led to a more significant decrease (*p* < 0.01). In UFibro, HC and MP were not able to reduce cell confluence over 12 d (Figure 1B,C).

As described above, free HC concentrations in plasma are 0.05–0.39 µg/mL and free MP concentrations are 0.001–1 µg/mL. Up to 80% of glucocorticoids bind to albumin and transcortin in plasma. With our cells remaining viable throughout the 12 d experiment, the following concentrations were chosen for all further experiments to stay as close as possible to in vivo parameters: 8 mM (=2.5 µg/mL) HC and 8 mM (=2.5 µg/mL) MP. Indeed, 20% of 2.5 µg/mL will be unbound in DMEM, yielding 0.5 µg/mL HC and 0.5 µg/mL MP that can interact with the cells. These concentrations are very close to the concentrations observed in vivo as described above.

**Figure 3:**
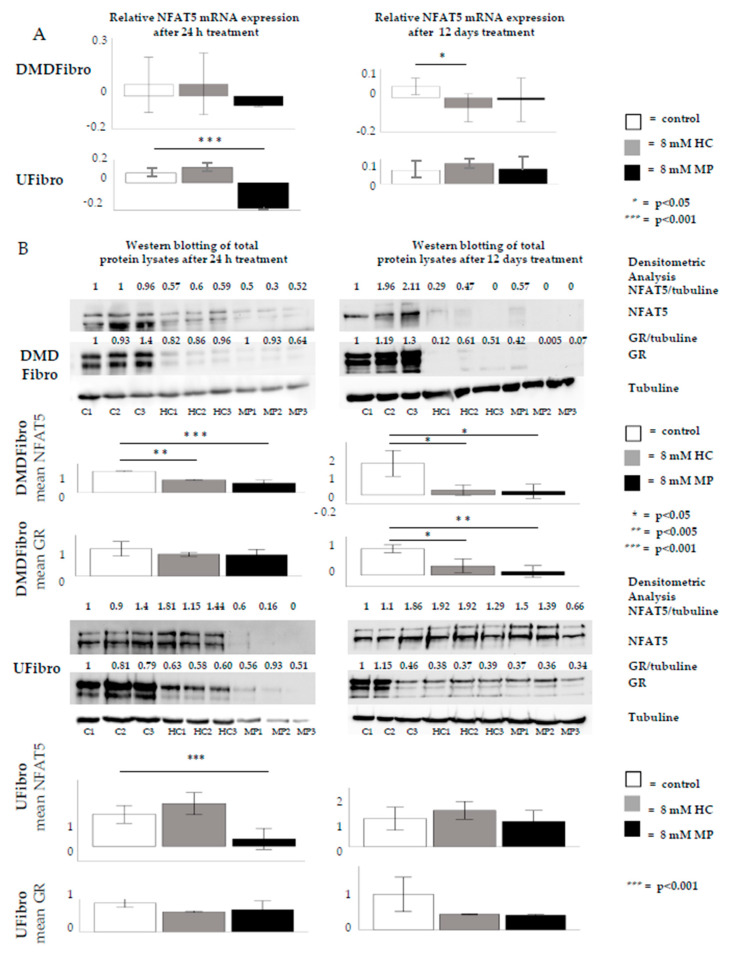
NFAT5 expression in DMD and unaffected fibroblasts treated for 12 d with hydrocortisone or methylprednisolone NFAT5 mRNA is significantly decreased in DMDFibro after 12 d treatment with 8 mM MP (*p* < 0.01) and UFibro after 24 h treatment with 8 mM MP (*p* < 0.001) (**A**). In Western blotting (WB), three passages are shown per condition (C1 = control passage 1, C2 = control passage 2, C3 = control passage 3, HC1 = hydrocortisone passage 1, HC2 = hydrocortisone passage 2, HC3 = hydrocortisone passage 3, MP1 = methylprednisolone passage 1, MP2 = methylprednisolone passage 2, MP3 = methylprednisolone passage 3). NFAT5 protein is significantly decreased in DMDFibro (*p* < 0.01 for HC and *p* < 0.001 for MP) after 24 h of 8 mM HC or 8 mM MP treatment. This decrease remains present after 12 days in both treatment conditions (*p* < 0.05). GR protein is significantly decreased in DMDFibro (*p* < 0.05 for HC and *p* < 0.01 for MP) after 12 days of 8 mM HC or 8 mM MP treatment. The only significant decrease in UFibro is present after 24 h treatment with 8 mM MP (*p* < 0.001) (**B**) (*n* = 3).

NFAT5 is an important protein in embryonic fibroblast growth. *NFAT5* expression was studied by RT-qPCR in DMDFibro. A significant downregulation in *NFAT5* was observed after 12 d in DMDFibro with 8 mM MP (Figure 3A). NFAT5 protein was measured semiquantitatively by Western blotting (WB) in DMDFibro and UFibro. After 24 h of 8 mM HC or 8 mM MP treatment in DMDFibro, a significant decrease in NFAT5 protein was observed (*p* < 0.01 for HC and *p* < 0.001 for MP), which remained decreased at day 12 in both conditions (*p* < 0.05). A significant decrease was also observed in UFibro treated for 24 h with 8 mM MP (*p* < 0.001). However, this decrease was not visible at day 12 in both treatment conditions (Figure 3B). GR protein expression was significantly decreased in DMDFibro treated for 12 d with 8 mM HC (*p* < 0.05) or 8 mM MP (*p* < 0.01; Figure 3B).

### 2.2. Insight into the Effect of Hydrocortisone and Methylprednisolone on DMD Fibroblast Growth through NFAT5-GR Interaction

Visualizing NFAT5 and GR at time point + 24 h in DMDFibro and UFibro treated with 8 mM HC or 8 mM MP was performed by confocal microscopy (CM). NFAT5 goat antibody was used [25]. All specificity tests were applied and valid (Supplementary Figures S1–S5).

**Figure 4:**
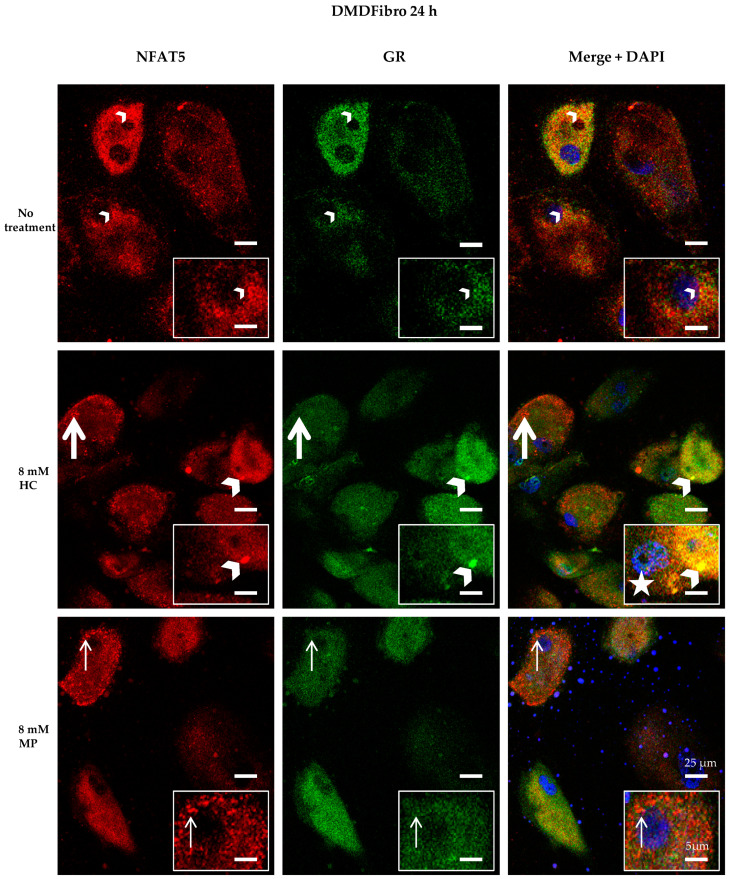
NFAT5 and GR localization in DMD fibroblasts treated for 24 h with hydrocortisone or methylprednisolone NFAT5 (red) and GR (green) are visualized by immunofluorescence. Nuclei are stained in DAPI (blue). Small yellow dots are seen in untreated DMDFibro (small white arrowheads). Here, both NFAT5 and GR are superimposed. Large yellow colocalization is only seen in DMDFibro with 8 mM HC (large white arrowheads). Orange dots are visible both after 8 mM HC and 8 mM MP treatment for 24 h, with small orange in 8 mM (small white arrow) and large ones in 8 mM HC (large white arrow). In orange dots, NFAT5 and GR are very close to each other but not superimposed. After 24 h with 8 mM HC, DMDFibro display very small yellow structures in the nucleus (white star), which stain for NFAT5 and GR (insets; *n* = 3).

**Figure 5:**
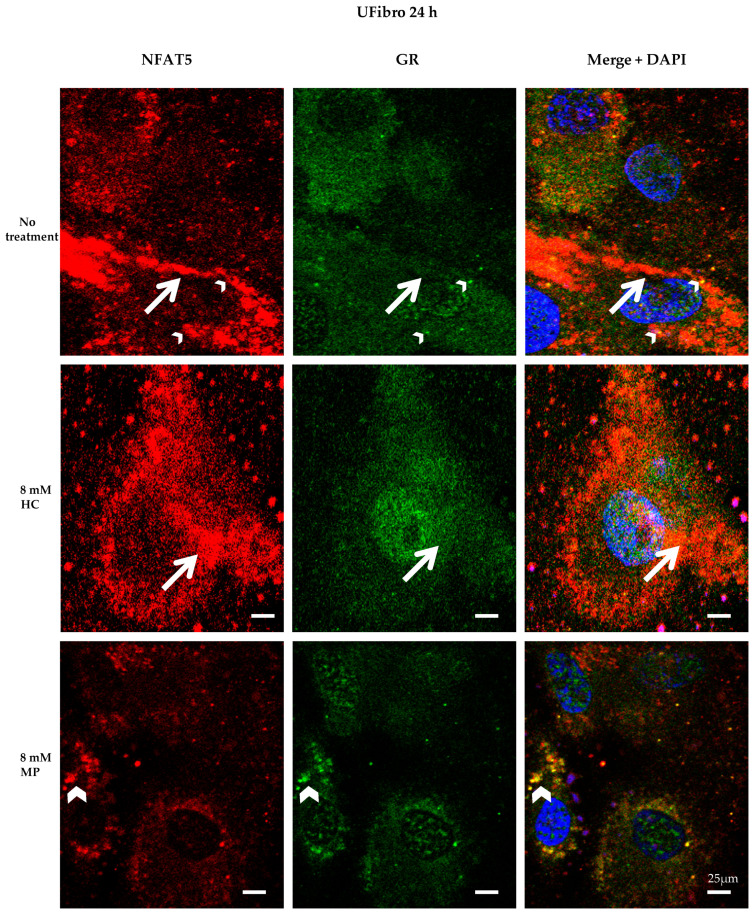
NFAT5 and GR localization in unaffected skeletal muscle fibroblasts treated for 24 h with hydrocortisone or methylprednisolone NFAT5 (red) and GR (green) are visualized by immunofluorescence. Nuclei are stained in DAPI (blue). Small yellow dots (small white arrowheads) and large orange dots (large white arrows) are seen in untreated UFibro or after treatment for 24 h with 8 mM HC. After 24 h with 8 mM MP, UFibro display large amounts of yellow round structures in the perinuclear area (large white arrowheads), which stain for NFAT5 and GR (*n* = 3).

In DMDFibro, treatment for 24 h with HC resulted in the emergence of large round structures in the near vicinity of the nucleus and in the cytoplasm, which stained both for NFAT5 and GR, with red and green staining being superimposed (Figure 4). Besides, small yellow dots were observed in untreated DMDFibro where both NFAT5 and GR were superimposed. After 24 h with 8 mM HC, DMDFibro showed very small yellow structures in the nucleus (white star), which stain for NFAT5 and GR. Orange dots were visible both after 8 mM HC and 8 mM MP treatment for 24 h, with small orange dots in 8 mM MP and large ones in 8 mM HC. In orange dots, NFAT5 and GR were very close to each other but not superimposed. In UFibro, small yellow dots and large orange dots were seen in the absence of treatment or treatment for 24 h with 8 mM HC. After 24 h with 8 mM MP, UFibro revealed large amounts of yellow round structures in the perinuclear area, which stained for NFAT5 and GR (Figure 5).

**Figure 6:**
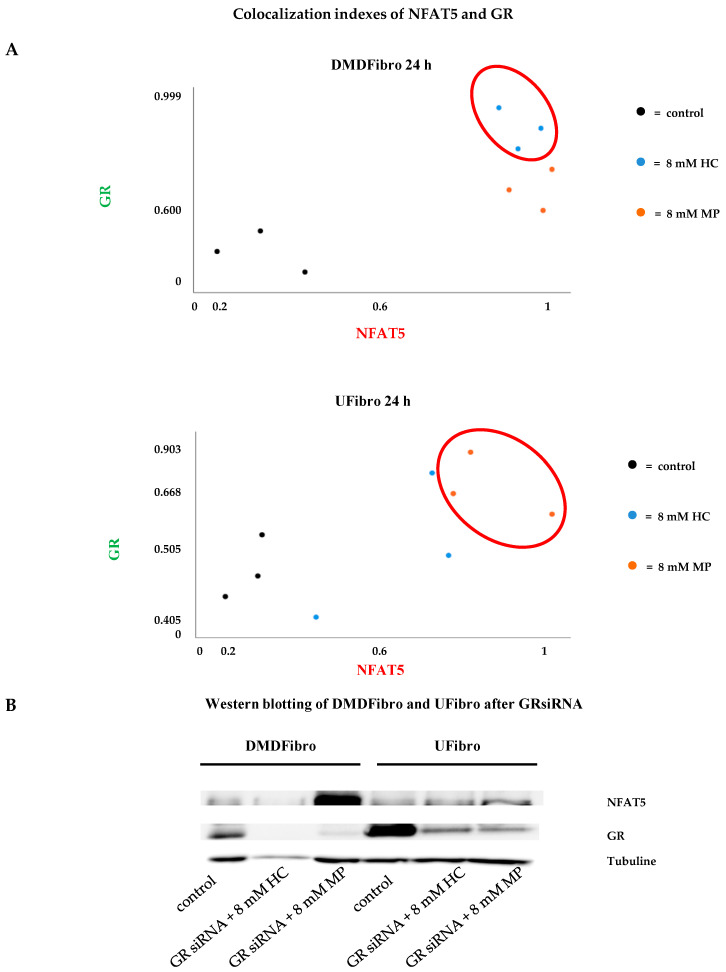
NFAT5 and GR colocalization indexes in DMD and unaffected skeletal muscle fibroblasts treated for 24 h with hydrocortisone or methylprednisolone and study of NFAT5 expression after GRsiRNA. Colocalization indexes were established by means of ImageJ. High indexes are observed in DMDFibro treated for 24 h with 8 mM HC and in UFibro treated with 8 mM MP for 24 h (*n* = 3) (**A**). After GRsiRNA in DMDFibro and UFibro, NFAT5 is expressed in both cell lines treated for 24 h with 8 mM HC or MP. GR is very weakly present after GRsiRNA compared to control cells (**B**).

Colocalization of NFAT5 with GR was further evaluated by ImageJ (Figure 6A). In DMDFibro, HC provided the highest colocalization indexes of NFAT5 with GR (Figure 6A, upper graph). This was only observed in UFibro treated for 24 h with 8 mM MP (Figure 6A, lower graph). By siRNA GR, the interaction between NFAT5 and GR was further validated. When GR was silenced, NFAT5 protein was present in the same amount as the controls on WB after 24 h treatment with 8 mM HC, both in DMDFibro and UFibro. In DMDFibro treated for 24 h with 8 mM MP, NFAT5 protein was even increased (Figure 6B).

## 3. Discussion

NFAT5 is known for modulating NF-κB under hyperosmolar conditions. More specifically, NFAT5- NF-κB complexes bind to the κB elements of NF-κB genes, thereby enhancing NF-κB activity [26]. The inhibition of NF-κB by GCs is a well described phenomenon. It occurs by induction of the IκB alpha inhibitory protein, trapping activated NF-κB in inactive cytoplasmic complexes [24,27]. In this work, only one culture of healthy skeletal muscle fibroblasts and one of DMD fibroblasts could be used. The COVID-19 pandemic abrogated further cell culture work. A larger study with more cell lines is planned. Here, DMD fibroblasts exposed for 24 h to HC and to a lesser extend MP showed NFAT5 colocalizing with GR in round structures located in the perinuclear area and the cytoplasm. In some DMD nuclei, similar structures were observed. These structures were also observed in unaffected skeletal muscle fibroblasts exposed for MP during 24 h. Interestingly, IκB masks the nuclear localization signal (NLS) of NF-κB, thereby blocking its nuclear translocation [28]. Localization of NFAT5-GR complexes in the cytoplasm could occur by masking of the NF-κB NLS, due to the presence of HC or MP induced IκB. With NFAT5 being merely located in the nucleus of DMD fibroblasts [22], GR could also bind to NFAT5 in the nucleus, inducing decreased cell growth over time. It could be conceivable after HC or MP treatment, DMD fibroblasts form NF-κB–NFAT-GR complexes in the cytoplasm and even the nucleus. In this study, a difference in DMD fibroblast confluence was noticed only after five days (120 h), whereas the control cells continued to grow for 12 d. This drop may be explained by the half-life of 10 h of NFAT5 [28,29]. The decrease in NFAT5 expression in unaffected skeletal muscle fibroblast after 24 h MP treatment was not noticed after 12 d treatment, possibly indicating to a cellular mechanism in healthy fibroblasts overcoming the NFAT5-GR binding. With NFAT5 being essential to fibroblasts survival, this mechanism could prevent fibroblasts from MP induced cell growth stop. However, treatment for 12 d with HC or MP revealed a significant decrease in GR in DMDFibro. The surgical left-over of the DMD patient was taken in a year where GCs intake by the patient is very likely. Differences in GCs sensitivity are a phenomenon also described in DMD [30]. If GR resistance is the underlying mechanism, which could explain this difference in sensitivity over time and GR interacting with NFAT5, an impact on NFAT5 expression could be expected in DMD fibroblasts. Indeed, if GR disappears over time, NFAT5 could re-emerge in DMD fibroblasts, thereby helping these cells regaining their fibrotic potential. This could be investigated further with longer cell culture times.

NFAT5 is also known for stimulating tumor growth factor TGF-β [31]. TGF-β is an important player in the formation of fibrosis [32], therefore a putative trapping of NFAT5 along with the well described downregulation of NF-κB in fibroblasts by HC and MP could explain the decreased fibroblast cell growth, which was observed. In DMD, prednisone therapy for 6 months results in significantly decreased endomysial connective tissue formation, the latter being mainly produced by fibroblasts [33]. The immunomodulatory effect of GCs is also well documented in DMD. These drugs have the ability to reduce the amount of mononuclear inflammatory cells and dendritic cells in DMD tissue [34]. However, the immune modulation cannot explain the entire range of effects of GCs in DMD [35]. MP is known for being 4 times relatively more potent than HC [36]. This was also observed in unaffected skeletal muscle fibroblasts at time point + 24 h with a significant decrease in NFAT5 protein expression after treatment with 8 mM MP, which was not observed after 8 mM HC. Appearance of round structures in the cytoplasm where NFAT5 and GR colocalized was noticed after treatment for 24 h with 8 mM MP. Hydrocortisone is equivalent to the naturally produced cortisol in the adrenal glands. The need for higher dosages of HC before an impact is noticed on NFAT5 could be a safeguard mechanism of the cell. Indeed, it would not be in the interest of fibroblasts to enter cell arrest after minor cortisone releases such as surgery or minor illnesses. At rest, adolescents produce 5–7 mg/m^2^/day of cortisol [37]. This production is increased by 3–5 times during surgery or minor illnesses, which returns to baseline within 24 h [38] and up to 10 times during severe trauma or surgery with up to 200–500 mg/day, which returns to baseline within 5 days [39]. It is not only important for cell viability that cortisol levels reenter physiological levels within days, also suppression of the hypothalamic-pituitary-adrenal (HPA) axis should be avoided in the body. DMD patients generally receive dosages of 0.75 mg/kg/day prednisolone, which averages 8 times the physiologic dose of hydrocortisone of 10 mg/m^2^/day, potentially leading to HPA axis disturbance [40] and could have an influence on other types of cells in the body. The 8 mM MP used in this study was within the range of plasma levels in DMD patients, meaning 8 times the physiological dosage of HC. When using 80 mM HC in this study, no decrease was observed in cell growth in unaffected skeletal muscle treated for 12 d with this dosage, possibly pointing to a safeguard mechanism in these cells to avoid loss of cell viability. However, at such dosages the HPA axis will be altered. The benefits of GCs usage in DMD should balance the downsides, which remains a physiological complex challenge.

In summary, treatment with 8 mM HC or 8 mM MP induced reduced cell growth in DMD fibroblasts, possibly due to colocalization of GR to NFAT5. Daily treatment with these GCs resulted in decreased NFAT5 and GR expression at 12 days. With NFAT5 being essential for fibroblast growth, these findings may help explain the influence of glucocorticoids in slowing down the process of fibrosis in DMD by modulating NFAT5 localization in the cell.

## 4. Materials and Methods

All methods were applied as described in our previous work [25].

### 4.1. Cell Lines

The Myobank Banque D’ADN (Paris, France) provided two primary fibroblast cell lines, obtained after patient’s consent. DMDFibro is a DMD fibroblast cell line originating from a DMD patient, male and 11 years old, from a surgical left-over from paravertebral muscle obtained during chirurgical treatment of scoliosis. UFibro are unaffected skeletal muscle fibroblasts donated by a healthy male donor, aged 17, from a surgical left-over of paravertebral muscle during scoliosis surgery. Both individuals were teenagers. More cell lines could not be cultured due to the COVID-19 pandemic.

Both cell lines were grown each in three different passages (*n* = number of passages; Supplementary Table S1) after Ethics Committee’s approval. DMEM contained all vital components and was supplemented with 10% FCS (Cambrex, Bioscience, Walkersville, MD, USA), penicillin (50 IU/mL, Gibco, Invitrogen, Carlsbad, CA, USA + streptomycin (50 mg/mL; Gibco), glucose and 1% L-glutamine (Life Technologies, Carlsbad, CA, USA) for healthy and germ free cell growth. Monthly checking for the presence of mycoplasma was performed with the MycoAlert Plus Kit (Lonza, Basel, Switzerland). All tests were negative for contamination during the entire experiment in both cell lines. Eurofins Genomics (Eurofins Scientific Group, Luxemburg, Luxemburg) identified both cell lines as explained in a previous work [22].

### 4.2. Cell Viability and Life Imaging by IncuCyte ZOOM

Fibroblasts seeded in low amounts (50 cells per cm^2^) shift toward the myofibroblast phenotype, which can be lost again in 3 d when seeded at high density (5.000 cells per cm^2^) [41]. Therefore, all seedings occurred at high density to conserve the fibroblast phenotype. DMDFibro and UFibro (*n* = 3) were seeded in a 24-NUNC well plate (Nalge Nunc International, Rochester, NY, USA; 4.000 cells/well, 1 mL/well and 1 well = 2 cm^2^) and incubated at 5% CO_2_ and 37 °C in the IncuCyte ZOOM (Essen Bioscience, Hertfordshire, UK) with real-time capturing of cell confluence over time. For 12 d, cells received daily addition of DMEM supplemented with following concentrations for HC (Solu-Cortef^®^, Pfizer, New York City, NY, USA) and MP (Solu-Medrol S.A.B.^®^, Pfizer): 80 nM (0.025 ng/mL), 8 µM (2.5 ng/mL), 80 µM (25 ng/mL), 8 mM (2.5 µg/mL) and 80 mM (25 µg/mL). Microscopic images (4 images/well) were taken every 2 h for the indicated time (24 h or 12 d). The IncuCyte Software (Essen Bioscience, Hertfordshire, UK) analyzed all data, resulting in cell confluency values. To determine the correct HC and MP dosages usable in cell cultures, cell viability was measured by CellTiter-Glo^®^ (Promega, Madison, WI, USA) luminescent cell viability assay following the supplier’s protocol. Briefly, this assay measured the presence of ATP produced by the cells at time point + 12 d and their viability by means of a bioluminescent reaction in the presence of luciferine and luciferase, expressed in relative light units (RLU).

### 4.3. Treatment with Methylprednisolone or Hydrocortisone

Optimal concentrations were established for HC and MP based on the results obtained from the growth curves and cell viability assays, and chosen in the ranges of free MP and HC concentrations in plasma as described above. DMEM was supplemented with 8 mM HC or 8 mM MP except for the controls, which received only DMEM. These HC and MP concentrations were used in cells seeded in 8-chamber slides (LabTek II, Nunc, Penfield, Hudson, MA, USA) for ICC studies or to 75-cm^2^ flasks for WB and RT-qPCR. All cell lines were treated for 24 h or 12 d with 8 mM HC or 8 mM MP.

### 4.4. RT-qPCR

DMDFibro and UFibro confluently grown in 75-cm^2^ flasks were exposed to 8 mM HC or 8 mM MP for 24 h and up to 12 d. Lysis needed a minimum purity of 1.9/2 measured by the A260/A280 ratio on BioDrop™ Touch Duo PC (Harvard Bioscience Inc., Holliston, MA, USA) [42]. RT-qPCR was carried out following minimum information for publication of quantitative real-time PCR experiments (MIQE) guidelines [43] and results were obtained by calculation with qBase+ Software version 2.6 (www.qbaseplus.com; Biogazelle, Zwijnaarde, Belgium) [44]. The 3 most stable reference genes were chosen from a set of tested candidate reference genes using geNorm (Supplementary Table S2).

### 4.5. Quantitative Western Blotting (WB)

Primary antibodies were applied to proteins from total protein extracts, which had been transferred to nitrocellulose membranes by electroblotting (Supplementary Table S3). Differences in protein concentration between samples were corrected using antitubulin (Sigma-Aldrich, St. Louis, MO, USA). Chemiluminescence (WesternBright™ Sirius, Advansta, Menlo Park, CA, USA) allowed one to visualize the immunoreactions under the Proxima 2650 (Isogen Life Science, De Meern, The Netherlands). Selectivity of the NFAT5 antibody was tested using siRNA NFAT5 (h): sc-43968 (Santa Cruz Biotechnology, Dallas, TX, USA) in DMDFibro as described earlier [25].

### 4.6. GR siRNA

Mechanistic insight into the interaction of GC with NFAT5 in fibroblast proliferation was partly provided by GR siRNA. By silencing GR gene expression, fibroblasts were not able to produce mRNA coding for GR. The lack of GR resulted in GC inactivity. By studying NFAT5 expression at time point + 24 h (WB) and comparing to fibroblasts without GR silencing, the influence of GC on NFAT5 was determined. GR silencing was performed as follows: 4 µL GR siRNA (h): sc-35505 (Santa Cruz Biotechnology,) was diluted in 2 mL serum free DMEM (=20 nM). The specificity of GR siRNA was determined by diluting 2 µL scrambled RNA (scRNA) in 2 mL serum free “DMEM”. Transfection was obtained by addition and incubation for 5’ at RT of 75 µL lipofectamine 2000 (ThermoFisher Scientific, Waltham, MA, USA) in 2 mL serum free DMEM. Subsequently, vials were added to siRNA and scRNA solutions followed by gentle mixing for 20′ at RT. Finally, flasks were incubated at 37 °C with 5% CO_2_ for 24 h.

### 4.7. Immunocytochemistry (ICC) and Confocal Microscopy (CM)

Staining procedures have been described in an earlier work [25]. Briefly, incubation during 1 h of primary antibodies was followed by another hour with secondary antibody to which AlexaFluor-488 (green) or AlexaFluor-555 (red) were attached (Invitrogen, Waltham, MA, USA). Antibodies and concentrations can be found in Supplementary Table S4. All specificity tests were performed in both cell lines: secondary antibody specificity with and without IgG Mouse or IgG Goat in the same concentrations as in the primary antibodies, siRNA GR and siRNA NFAT5. Positive controls were performed in unaffected myoblasts [25].

Confocal images were captured with a Zeiss LSM880 confocal microscope and its 40×Plan-Apochromat/1.3 oil objective, where the pinhole was set at 1Airy Unit (Zeiss, Jena, Germany). Colocalization indexes M1 (NFAT5) and M2 (GR), Pearson’s correlation coefficient (*r*) and Mander’s Overlap coefficient (R) and were measured with Image J [25,45].

### 4.8. Statistical Analysis

By means of SPSS 27.0 (IBM, Armonk, NY, USA), mean and standard deviation were obtained for each cell line passage at each time point and for each condition: control, 8 mM HC and 8 mM MP. By using one-way ANOVA with Tukey’s multiple comparison test, means and standard deviations from three biological replicates led to one mean and one standard deviation, considering: * = *p* < 0.05, ** = *p* < 0.01 and *** = *p* < 0.005.

## Supplementary Materials

Supplementary materials can be found at https://www.mdpi.com/1422-0067/21/23/9225/s1.

## Abbreviations

CBG	corticoid binding globulin
DAG	donkey anti goat
DAM	donkey anti mouse
DMD	Duchenne muscular dystrophy
DMDFibro	Duchenne muscular dystrophy fibroblasts
DMEM	Dulbecco’s modified Eagle medium
ECM	extracellular matrix
FITC	fluorescein isothiocyanate
GCs	glucocorticoids
GR	glucocorticoid receptor
HC	hydrocortisone
HCSS	hydrocortisone sodium succinate
HPA	hypothalamic-pituitary-adrenal
ICC	immunocytochemistry
IM	intramuscular
IV	intravenous
MIQE	minimum information for publication of quantitative real-time PCR experiments
MP	methylprednisolone
MPSS	methylprednisolone sodium succinate
N	number of passages
NFAT5	nuclear factor of activated T-cells 5
NF-κB	nuclear factor kappa-light-chain-enhancer of activated B cells
RLU	relative light units
RT-qPCR	real-time quantitative PCR
scRNA	scrambled RNA
siRNA	silencing RNA
UFibro	unaffected skeletal muscle fibroblasts
UMyo1	unaffected myoblasts
WB	Western blotting
