# Supplementary material for: Description of a Novel Mechanism Possibly Explaining the Antiproliferative Properties of Glucocorticoids in Duchenne Muscular Dystrophy Fibroblasts Based on Glucocorticoid Receptor GR and NFAT5
Source: Int J Mol Sci. 2020 Dec 3;21(23):9225. doi: 10.3390/ijms21239225 (PMC7731298; doi:10.3390/ijms21239225)
Supplement: Supplementary file 1 [file ijms-21-09225-s001.pdf]

Supplementary Materials:

Supplementary Table 1. Cell culture data.

Primary fibroblast culture	Status	Provided by	Age (years)	Gender	Mutation/ Deletion
UFibro	Healthy	Myobank (F)	17	Male	NA
DMDFibro	DMD	Myobank (F)	11	Male	Exon 49-50

DMD= Duchenne muscular dystrophy.

Supplementary Table 2. Genes used in RT-qPCR.

Gene	Primer	Concentration	Source
NFAT5	PrimePCR SYBRGreen Assay qHsaCID0015734 intron-spanning	1x	Bio-Rad
UBC	F:ATTTGGGTCGCGGTTCTTG	1,25pmol	IDT
	R:TGCCTTGACATTCTCGATGGT	1,25pmol	IDT
HPRT1	F: TGACACTGGCAAAACAATGCA	1,25pmol	IDT
	R: GGTCCTTTTCACCAGCAAGCT	1,25pmol	IDT
B2M	F:TGCTGTCTCCATGTTTGATGTATCT	1,25pmol	IDT
	R: TCTCTGCTCCCCACCTCTAAGT	1,25pmol	IDT
RPL13A	F:CCTGGAGGAGAAGAGGAAAGAGA	1,25pmol	IDT
	R:CCTGGAGGAGAAGAGGAAAGAGA	1,25pmol	IDT
YWHAZ	F:ACTTTTGGTACATTGTGGCTTCAA	1,25pmol	IDT
	R: CCGCCAGGACAAACCAGTAT	1,25pmol	IDT
SDHA	F: TGGGAACAAGAGGGCATCTG	1,25pmol	IDT
	R: CCACCACTGCATCAAATTCATG	1,25pmol	IDT
HMBS	F: GGCAATGCGGCTGCAA	1,25pmol	IDT
	R: GGGTACCCACGCGAATCAC	1,25pmol	IDT
TBP	Unknown	1,25pmol	[27]
AluSq	Unknown	1,25pmol	[27]
AluSx1	Unknown	1,25pmol	[27]

[UBC]= ubiquitin C; [HPRT1]= hypoxanthine phosphoribosyltransferase 1; [B2M]= beta-2 microglobulin; [RPL13A]= 60S ribosomal protein L13a; [YWHAZ]= Tyrosine 3-Mono-oxygenase/Tryptophan 5-Mono-oxygenase Activation Protein, Zeta; [SDHA]= Succinate dehydrogenase complex, subunit A, [HMBS]= hydroxymethylbilane synthase, [TBP]= TATA-binding protein, [AluSq]= Alu restriction enzym, [AluSx1]= interspersed repeat subfamily. [NFAT5]= Nuclear Factor of Activated T-cells 5.

Supplementary Table 3. Primary antibodies used in Western-blotting.

Antigen	Primary antibody	Clone	Concentration	Source

Tubuline	Mouse monoclonal Ig G1	B-5-1-2	1/1000	Sigma-Aldrich
NFAT5	Mouse monoclonal IgG2a	F-9	2µg/mL	Santa-Cruz Biotechnology

NFAT5= Nuclear Factor of Activated T-cells 5.

Supplementary Table 4. Primary antibodies used in immunocytohistochemistry.

**Antigen**	**Primary antibody**	**Clone**	**Concentration**	**Source**
NFAT5 (V-18)	Goat polyclonal IgG	/	10 µg/mL	Santa-Cruz Biotechnology

NFAT5= Nuclear Factor of Activated T-cells 5; NCAM= neural cell adhesion molecule.

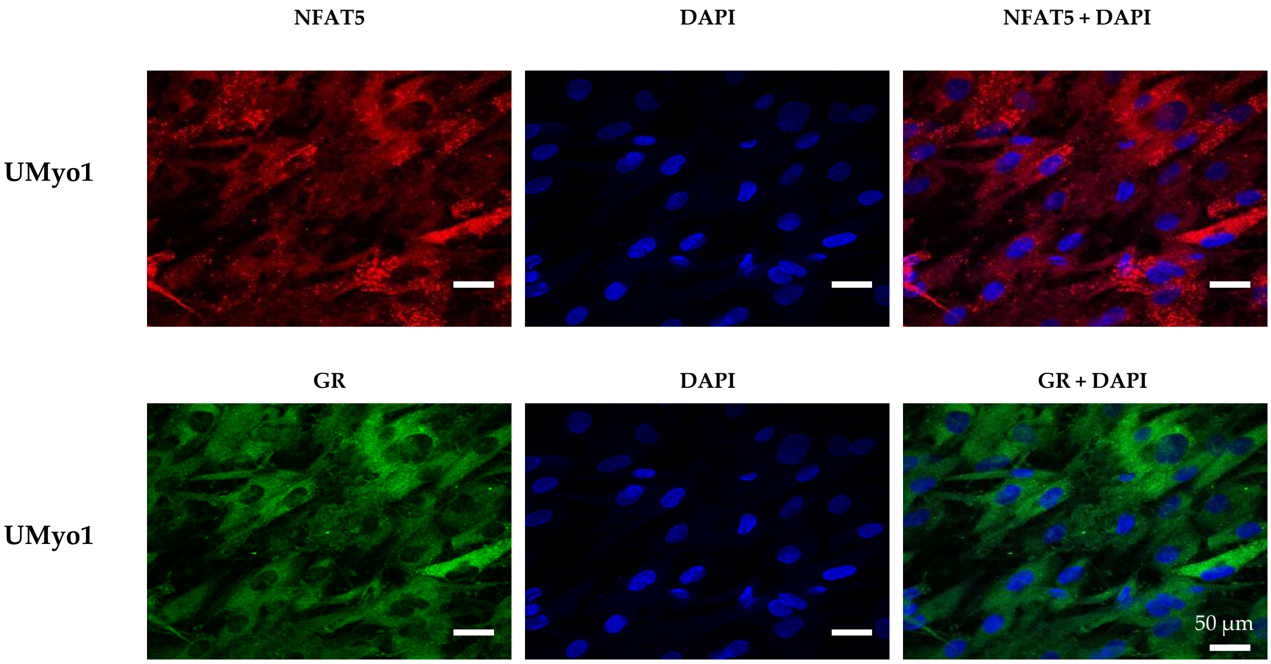

Fig. 1: NFAT5 and GR localization in unaffected myoblasts UMyo1 NFAT5 (red) and GR (green) are visualized by immunofluorescence. Nuclei are stained in DAPI (blue) (n=3). UMyo1 have been extensively used in a previous work. GR is clearly visible as a green staining (n=3).

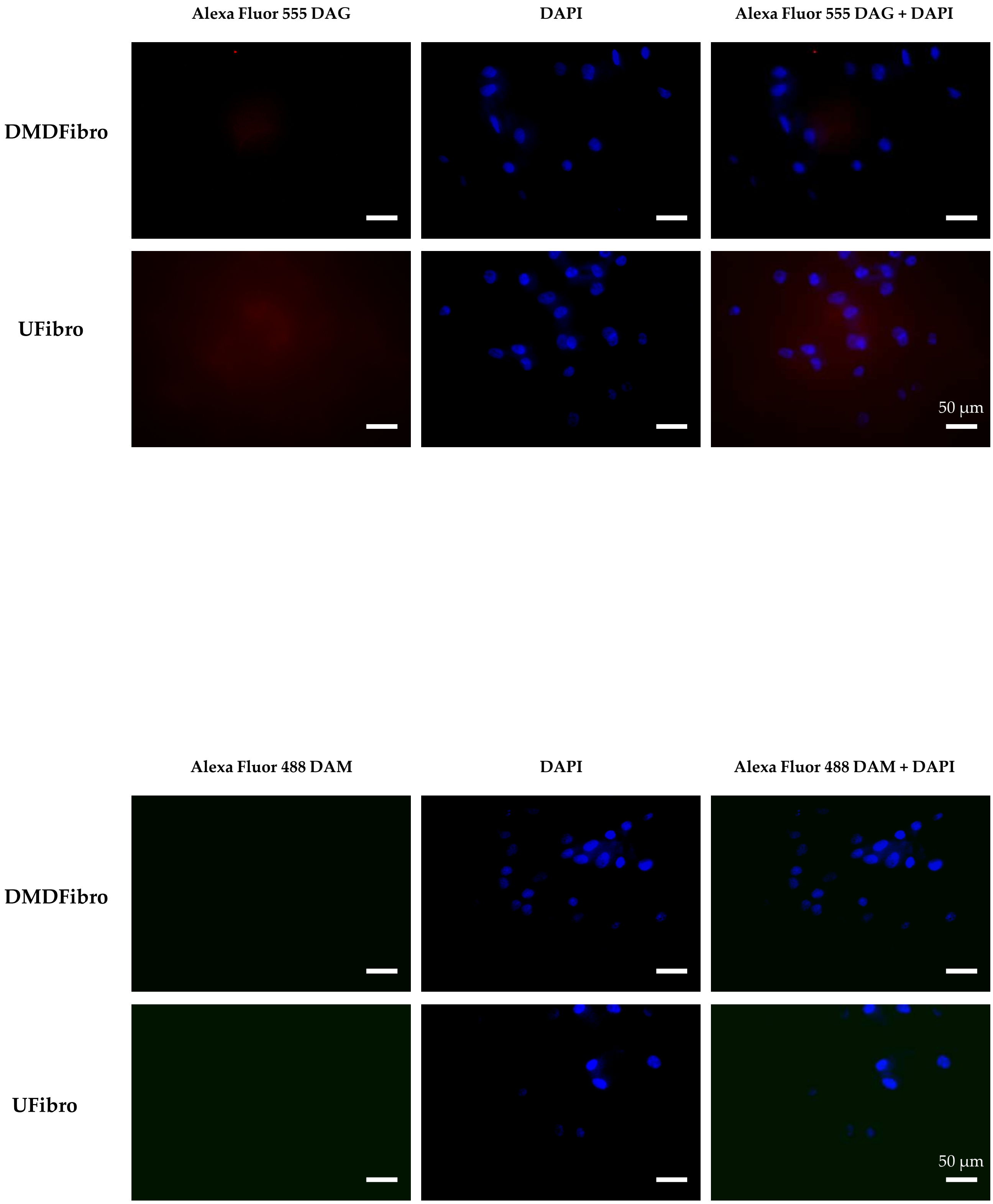

Fig. 2: Testing of secundary antibodies in DMD and unaffected skeletal muscle fibroblasts Alexa Fluor 555 donkey anti goat (DAM) (red) and Alexa Fluor 488 donkey anti mouse (DAM) (green) are visualized by immunofluorescence. Nuclei are stained in DAPI (blue). No staining visible in red and in green channels, pointing to absence of aspecific binding by secundary antibodies (n=3).

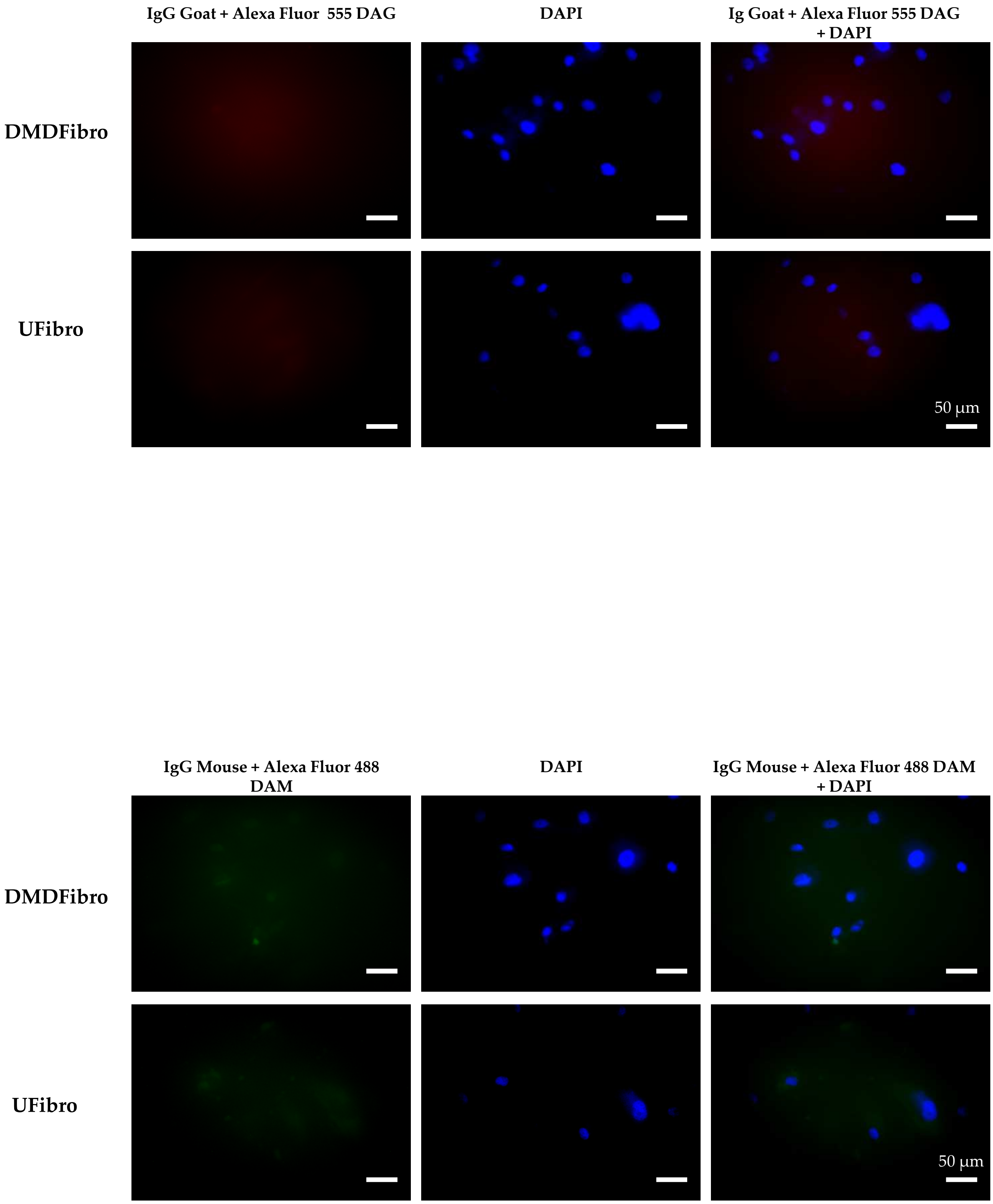

Fig. 3: Testing of IgG Goat and IgG Mouse in DMD and unaffected skeletal muscle fibroblasts IgG Goat (red) used in the same concentration as the NFAT5 antibody and Ig G Mouse (green) used in the same concentration as the GR antibody are visualized by immunofluorescence. Nuclei are stained in DAPI (blue). No staining visible in the red channel and minor staining in the green channel, pointing to absence of aspecific binding of animal IgG in which the antibodies were produced and harvested (n=3).

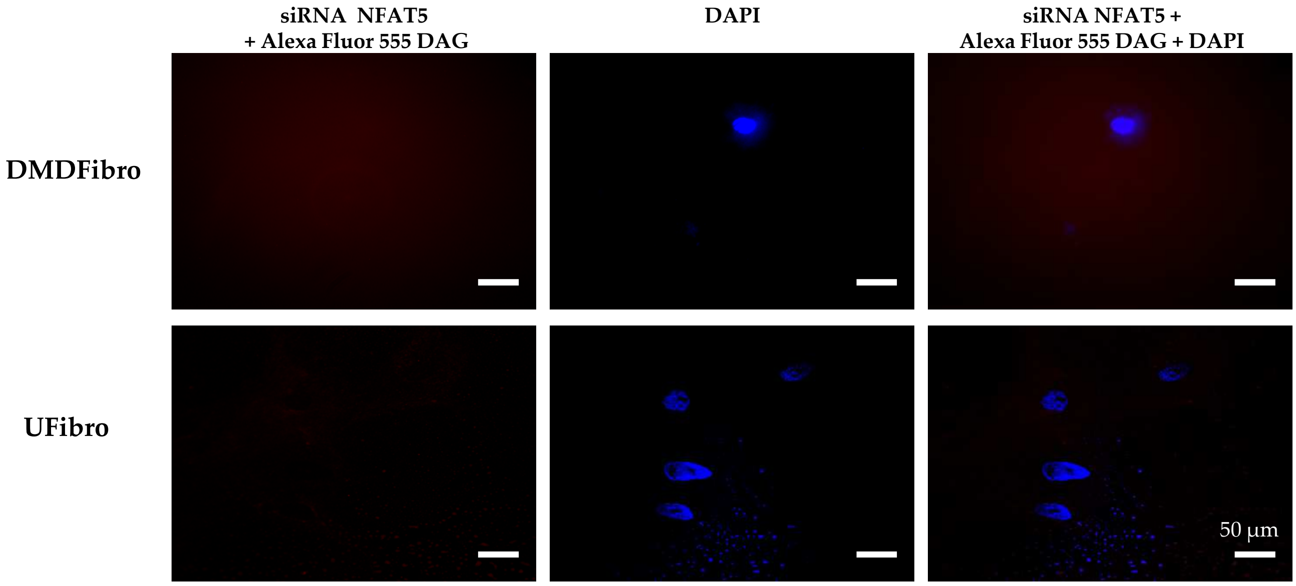

Fig. 4: Testing of NFAT5 Goat in DMD and unaffected skeletal muscle fibroblasts NFAT5 (red) is visualized by immunofluorescence after NFAT5 siRNA. Nuclei are stained in DAPI (blue). No staining visible in the red channel, pointing to absence of aspecific binding of NFAT5 Goat in DMD and unaffected skeletal muscle fibroblasts (n=3).

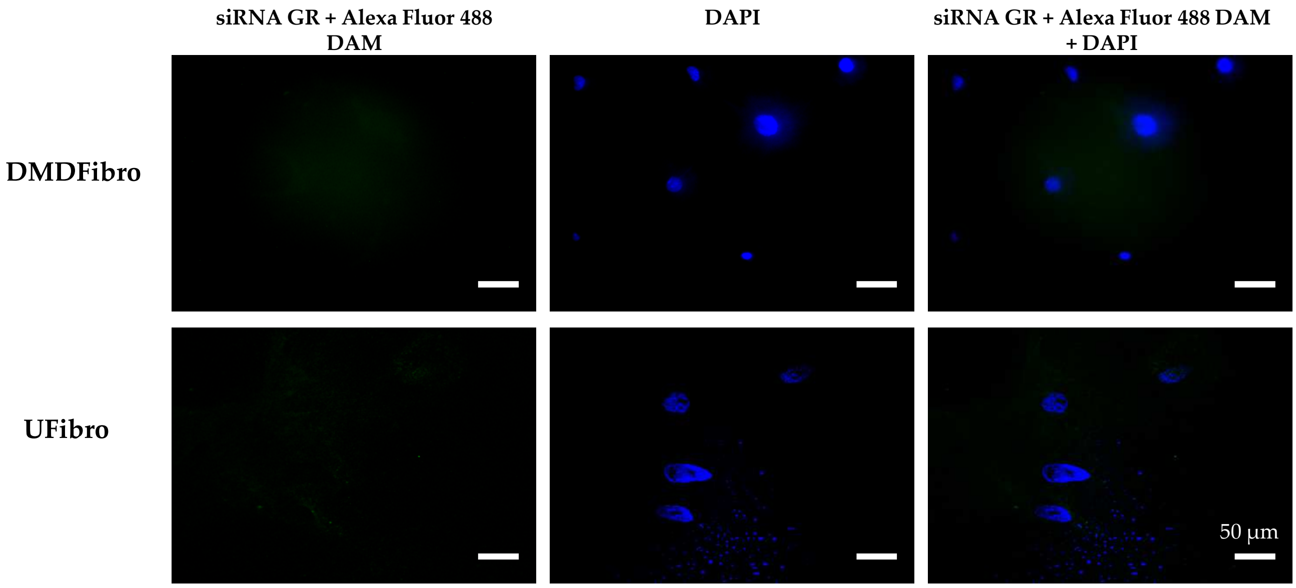

Fig. 5: Testing of GR Mouse in DMD and unaffected skeletal muscle fibroblasts GR (green) is visualized by immunofluorescence after GR siRNA. Nuclei are stained in DAPI (blue). No staining visible in the green channel, pointing to absence of aspecific binding of GR Mouse in DMD and unaffected skeletal muscle fibroblasts (n=3).
